# Level of IL-6, TNF, and IL-1β and age-related diseases: a systematic review and meta-analysis

**DOI:** 10.3389/fimmu.2024.1330386

**Published:** 2024-03-01

**Authors:** Anna Tylutka, Łukasz Walas, Agnieszka Zembron-Lacny

**Affiliations:** ^1^ Department of Applied and Clinical Physiology, Collegium Medicum University of Zielona Gora, Zielona Gora, Poland; ^2^ Institute of Dendrology, Polish Academy of Sciences, Kórnik, Poland

**Keywords:** comorbidities, immunosenescence, interleukin 1β, interleukin 6, tumor necrosis factor

## Abstract

**Introduction:**

Chronic low-grade inflammation is an important aspect of morbidity and mortality in older adults. The level of circulating pro-inflammatory cytokines (interleukin (IL)-6, tumor necrosis factor (TNF) or IL-1β) is a risk factor in cardiovascular and neurodegenerative diseases and is also associated with sarcopenia and frailties. The objective of this study was to assess each cytokine: IL-6, TNF, and IL-1β separately in the elderly with comorbidities against controls without diseases according to the data published in the available literature.

**Methods:**

The electronic bibliographic PubMed database was systematically searched to select all the relevant studies published up to July 2023. The total number of the subjects involved in the meta-analysis included patients with diseases (*n*=8154) and controls (*n*=33967).

**Results:**

The overall concentration of IL-6 was found to be higher in patients with diseases compared to controls and the difference was statistically significant, with a *p*-value of <0.001 (SMD, 0.16; 95% CI, 0.12–0.19). The heterogeneity was considerable with Q = 109.97 (P <0.0001) and I^2^ = 79.2%. The potential diagnostic usefulness of IL-6 was confirmed by odds ratio (OR) analysis (OR: 1.03, 95% CI (1.01; 1.05), *p*=0.0029). The concentration of both TNF and IL-1β was elevated in the control group compared to patients and amounted to SMD -0.03; 95% CI, -0.09–0.02, *p*-value 0.533 and SMD-0.29; 95% CI, -0.47– -0.12; *p* = 0.001, respectively. For TNF, however, the difference was statistically insignificant.

**Discussion:**

IL-6, unlike TNF and IL-1β, could be a useful and convenient marker of peripheral inflammation in older adults with various comorbidities.

## 1 Introduction

In developed countries, a trend towards a shift in the age structure towards the elderly population can be observed, which can have health care as well as economic consequences. According to the latest estimates, the number of older people over 60 will double from 756 to 1,400 millions by 2030 (1). The aging of the immune system is a paradox of immunosenescence (deficiencies) and excessive inflammation (inflammaging), causing immune disorders. Inflammaging described as a chronic systemic inflammation associated with chronic age. The changes are mainly attributed to the somatic senescence-associated secretory phenotype (SASP) (2). It has been proposed that the basal inflammatory state and aging is a major factor in the increasing incidence of chronic diseases such as cardiovascular, neurodegenerative, and metabolic diseases (3). The coexisting multimorbidity in the elderly increases the risk of multiple organ failure and death. As the aging of the immune system progresses, the elderly become more susceptible to infectious diseases and cancer, and the risk of dying of influenza and coronavirus disease (COVID-19) also increases (4). It is therefore clear that there is an interplay between the aging of the immune system and age-related diseases. Solutions to prevent and treat age-related diseases can be sought through analyses of both the inflammation severity and immunosenescence (5).

Despite many features of acute inflammation, chronic inflammation is usually low-grade and results in tissue degeneration. There are several mechanisms which activate the development of chronic inflammation, which has been described as the following theories: 1) stress theory, which emphasizes the role of the correlation between excessive stress reaction and stronger pro-inflammatory response in the elderly (6); 2) the theory of oxidation-inflammation, according to which oxidative stress leads to inflammatory aging and affects homeostasis and prevention of health (7); 3) DNA damage theory, where damage to DNA telomeres and mitochondrial DNA leads to point mutations and chromosome rearrangements that contribute to cell aging (8); 4) theory of stem cells aging, where chronic inflammation is one of the main factors responsible for stem cells aging (9); and most widely discussed 5) theory of cytokines, where pro-inflammatory cytokines play an important role in inflammatory aging caused by chronic inflammation.

Aging facilitates a pro-inflammatory state by disrupting the peripheral immune system, which leads to excessive innate immune activity with the release of pro-inflammatory cytokines and a decrease in anti-inflammatory cytokines (10, 11). Different pro-inflammatory cytokines, such as interleukins (IL): IL-1β, IL-6, IL-12, IL-18, interferon (IFN-γ), and tumor necrosis factor (TNF), as well as anti-inflammatory ones, such as IL-4, IL-10, IL-13, and IL-19 which are secreted from immune cells, interact with body cells to mediate the immune responses and thus elicit its most optimum outcome (12–15). Elevated levels of interleukin-6 and TNF (16), as well as IL-1β (17), are associated with diseases, disability, and mortality in older adults. Interleukin-6, also known as ‘the cytokine for gerontologists’, plays a key role in the acute phase response in metabolic control and in the pathogenesis of many chronic diseases (18). IL-6 is produced mainly by the monocytes and macrophages (19). It produces a pleiotropic effect, and although in healthy and younger people its level is usually relatively low, in the elderly its elevated levels may correlate with increased mortality (20–24). Both Sánchez-Castellano et al. (25) and Mehta et al. (26) showed an association between inflammation, including IL-6 levels, and sarcopenia severity. A meta-analysis by Ng et al. (11), in which 34 studies were analyzed, demonstrated that the elderly with depression had significantly higher peripheral levels of IL-1β (*p*=0.026) and IL-6 (*p*<0.001) which was not the case in TNF (*p*=0.351) and C-reactive protein (CRP) levels (*p*=0.05). IL-1β is a key mediator of inflammatory response and participates in cell proliferation, differentiation, and apoptosis (27). This cytokine is expressed by a wide variety of cells, but macrophages and monocyes are particularly important, where IL-1β is produced in large quantities during infections and other stressful events (28, 29). Overproduction of IL-1β has also been confirmed in major depressive disorder, which may be related to the inflammasome hypothesis (27). High glucose concentration was reported to stimulate the production of IL-1β by pancreatic β cells, which implies the role of this cytokine also in type 2 diabetes (30–32). Since type 2 diabetes in the elderly increases the risk of cardiovascular disease, blocking IL-1β in these patients may reduce the incidence of myocardial infarction and stroke (31). Similar observations were recorded by Alzamil (33) in patients with obesity and with type 2 diabetes, where serum TNF levels in patients with diabetes and obesity were significantly higher than in patients without obesity (*p*<0.018). Significantly higher serum TNF levels were detected in patients with diabetes and obesity than in non-diabetic group with obesity (p<0.001). TNF is a pro-inflammatory mediator that can produce beneficial effects when activated locally in the tissues but it can be highly harmful when released systemically. It is one of the most important cytokines, produced by several types of cells: monocytes, T-cells, macrophages, fibroblast, adipocytes and smooth muscle cells (34). Generally, TNF is released together with interleukins usually IL-1 (35). In elderly people and centenarians, it has been shown that the level of TNF rises, which significantly increases mortality. Moreover, TNF has been shown to mediate metabolic changes and increased TNF levels have been found in patients with type 2 diabetes (24).

The assessment of chronic inflammation, including the level of pro-inflammatory cytokines in elderly people with comorbidities, may be the key to more effective treatment. Our hypothesis was that independent measurements of cytokines such as IL-6, TNF and IL-1β were significantly associated with the development of age-related diseases. Therefore, the aim of this study was to independently evaluate three cytokines: IL-6, TNF and IL-1β in elderly people with comorbidities compared to disease-free controls.

## 2 Materials and methods

### 2.1 Search strategy and study selection

This systematic literature review and meta-analysis followed the requirements of the PRISMA statement (36). We comprehensively searched the Internet literature in the PubMed/Medline database. We focused on scientific papers that contained the word “cytokines” and at least one of the cytokines: IL6 or/and TNF alpha or/and IL-1 beta, and one of the following terms: “cut off” or/and “odds ratio”. Next, we excluded all the papers that involved training or therapy, as such interventions could affect the cytokine levels. Finally, the following filters were also used to obtain our working database: papers in English, free full text available, and study patients’ age above 65. The queries were last updated on 14 August 2023, and **Table 1** presents the search strategy using MeSH terms. Detailed search code attached in the **Supplementary Material File**.

**Table 1:** Search strategy for PubMed/Medline.

Search #	Search Strategy	Items Found
1	cytokines	968.547
2	(IL-6) OR (Interleukin 6)	184.784
3	(TNF alpha) OR (Tumor necrosis factor alpha)	263.299
4	(IL-1 beta) OR (Interleukin 1 beta)	63.155
5	(cut off) OR (Odds ratio)	434.548
6	(#2) OR (#3) OR (#4)	387.590
7	(#1) AND (#5)	9.712
8	(#7) AND (#6)	3.7999
9	training	2.553.838
10	therapy	11.214.058
11	(#8) NOT (#9)	3.623
12	(#11) NOT (#10)	2.504
	Filtres	
	English	2.439
	Free full text	1.070
	Age 65+	371

### 2.2 Inclusion and exclusion criteria

The following eligibility requirements were applied: (1) the participants of the study were elderly, additionally filtered in PubMed by the search terms: ‘aged: 65+ years’ (2) the older adults in the study design were clearly divided into two groups: the group with a disease entity and without diseases as the control group; (3) cytokine levels were measured quantitatively; (4) sufficient information and data were provided to estimate the mean value and standard deviation (SD). Reviewers independently evaluated the eligibility of the research papers found, and any disagreement was resolved through discussion. The exclusion criteria were as follows: (1) duplicate publications; (2) reviews, meta-analyses, protocols, editorials, letters, preprints, and unavailable full texts; (3) studies in which a healthy control group was missing or another disease entity was selected as the control group; (4) studies in which interventions were described, i.e. the impact of treatment (therapy), diet, physical activity; (5) studies on animals, (6) non-English research papers.

### 2.3 Data extraction and quality assessment

Each paper was annotated using the following data: the name of first author and the year of publication, the number of included individuals with respect to sex in both study and control groups, the age of study and control groups, IL-6, TNF and IL-1β level, and ratio (OR) values (if applicable in the single model).

### 2.4 Statistical analysis

Statistical analyses were performed using R 4.2.1 software (37), and a “meta” package (38) using a random effects model. For statistical homogeneity, medians and IQRs were converted to means with *SDs* to maximize the number of studies eligible for meta-analysis (39). Chi-squared and Higgin’s I^2^ tests were used to measure heterogeneity between studies. Cut-off values of 25%, 50%, and 75% were applied to label heterogeneity as low, moderate, or high respectively (40). Heterogeneity between studies was tested using Standardized Mean Difference (SMD) and “metacont” function, whereas studies with OR were analyzed using “metagen” function. Funnel plots were used to assess publication bias. Asymmetry was present in the funnel plots, Egger’s and Begg’s tests were applied to quantitatively assess whether there was any publication bias. The significance threshold for all statistical tests was set at *p* < 0.05.

## 3 Results

### 3.1 Study search and characteristics

**Figure 1:**
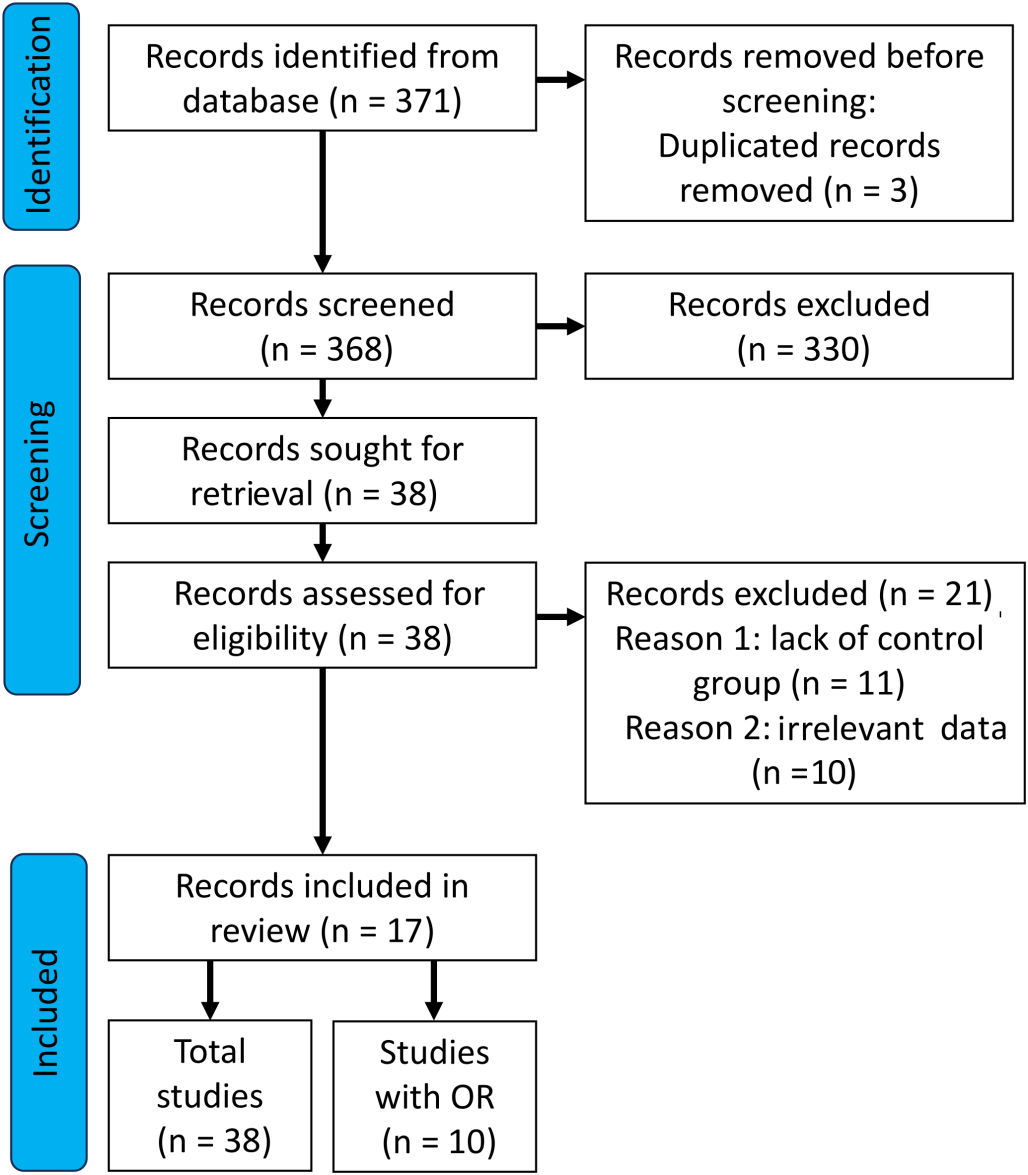
Preferred Reporting Items for Systematic Reviews and Meta-Analyses (PRISMA) flow diagram showing the paper selection process for inclusion.

A detailed selection of studies which were included in our analysis is shown in **Figure 1**. The initial search yielded 371 records. After removing duplicates (*n*=3), 368 studies were screened. After reviewing the abstracts and titles of the manuscripts, we excluded a total of 330 records from further analysis. Thirty eight publications (records) were qualified for the next stage, of which *n* = 21 records were removed. The reasons for deleting the publication was: *n*=11 lack of control group and *n*=10 irrelevant data - data presented in the form of unadjusted and adjusted models. Finally, *n*=17 publications were assessed. Due to the fact that some of the publications included more than one tested group, the total number of studies was *n*=38. For OR analyses, *n*=6 publications were included, and the total number of studies was *n*=10.

Publication dates of the included studies ranged from 2008 to 2023. All the included studies reported the measurement of cytokine levels quantified in patients/controls blood and serum or plasma. The total number of the included subjects in the meta-analysis equaled n=8154 patients with diseases and *n*=33967 controls. With regard to IL-6 concentration investigation,15 publications were included, five of which were used twice in the analysis (41–45) because they involved two groups of patients with a disease and one control group. In turn, the research conducted by Sánchez-Castellano et al. (25) was used three times in IL-6 level assessment (as there were three groups of patients with sarcopenia described by different criteria). Nine publications were included in the evaluation of TNF level, one of which i.e., the research by Sánchez-Castellano et al. (25), was used three times and the research conducted by Jefferis et al. (41) was used twice. Two publications were investigated for IL-1β evaluation and the research carried out by Sánchez-Castellano et al. (25) was used three times (**Table 2**). For the analysis of the OR for IL-6, five manuscripts were selected where the OR values ​​were publicly available and were reported as 95% Cl. The publications by Tattersall et al. (44) and Wennberg et al. (52) were used twice because two independent models were applied in the study design. The OR analysis for TNF-α included two manuscripts: Wennberg et al. (52) and Anaszewicz et al. (54). OR analysis was not performed for IL-1β due to a lack of data (**Table 3**).

**Table 2:** The list of publications included in this meta-analysis based on SMD.

Study		Control				Disesase				
	Patients Nationality	N	Age (years)	% of women	Levels	Type of disease	N	Age (years)	% of women	Levels
IL-6 (pg/ml) studies										
25 (I)	Spanish	133	87.4 ± 5.3	80.2	12.2 ± 10.3	Sarcopenia (Janssen)	17	87.4 ± 5.3	70.6	15.3 ± 12.7
25 (II)	Spanish	98	87.8 ± 5.0	76.8	12.8 ± 9.3	Sarcopenia (Masanés)	52	87.7 ± 4.9	82.4	12.3 ± 12.9
25 (III)	Spanish	9	86.2 ± 6	70	10.8 ± 5.5	Sarcopenia (low handgrip EWGSOP2)	140	87.8 ± 4.8	79.9	12.6 ± 10.9
46	American	139	61.8 ± 7.6	2.9	0.85 ± 0.73	Barrett’s esophagus	141	62.8 ± 6.7	2.1	1.01 ± 0.92
47	American	925	66 ± 11	19	2.52 ± 1.84	Atrial Fibrillation	46	74 ± 10	6	3.76 ± 2.00
48	American	972	50-79	100	1.8 ± 1.4	Ischemic stroke	972	50-79	100	2.1 ± 1.9
41 (I)	British	698	70.80 ± 5.44	26.9	2.43 ± 1.43	Myocardial infarction	362	70.91 ± 5.48	26.4	2.99 ± 1.76
41 (II)	British	587	71.35 ± 5.24	36.1	2.56 ± 1.40	Stroke	299	71.29 ± 5.32	36.6	2.83 ± 1.61
26	American	622	71.0 (66.0-77.0)	32.5	1.65 ± 1.08	Liver Disease	586	68.0 (64.0-74.0)	80.2	1.58 ± 0.96
44 (I)	American	4532	61.8 ± 10.0	51.7	1.52 ± 1.21	Intermittent asthma	388	59.7 ± 10.1	60.8	1.60 ± 1.21
44 (II)	American	4532	61.8 ± 10.0	51.7	1.52 ± 1.21	Persistent asthma	109	63.4 ± 9.9	70	1.89 ± 1.61
49	Korean	68	67.6 ± 11.2	35.3	10.5 ± 17.5	Acute exacerbation	68	70.3 ± 5.8	32.3	35.5 ± 68.3
45 (I)	American	5674	65+	58.2	2.2 ± 1.9	Incident Parkinson's Disease	154	65+	45.4	2.2 ± 2.2
45 (I)	American	5674	65+	57.8	2.2 ± 1.9	Prevalent Parkinson's Disease	60	65+	38.3	2.4 ± 1.9
50	American	643	65.3 ± 9.2	48.1	1.5 ± 1.3	Diabetic retinopathy	278	65.0 ± 9.2	47.9	1.5 ± 1.4
51	American	616	85.3 ± 3.3	59	3.25 ± 1.98	Cardiovascular disease	434	86.4 ±4.0	70	3.81 ± 2.80
43 (I)	Italian	30	62.3 ± 50-74	30	0.89 ± 1.41	Hepatocellular carcinoma	30	62.3 (50-74)	30	22.19 ± 21.64
43 (II)	Italian	30	62.3 (50-74)	30	0.89 ± 1.41	Liver cirrhosis	30	62.3 (50-74)	30	5.47 ± 5.76
42 (I)	American	296	65.2 ± 10.4	50	2.1 ± 1.83	Lung cancer NCI-MD study	270	66.6 ± 10.0	47.4	3.7 ± 3.65
42 (II)	American	595	64.5 ± 5.3	36.1	4.0 ± 2.90	Lung cancer PLCO study	532	64.7 ± 5.1	32.5	4.4 ± 3.20
52	American	1416	71.9 (63.6-78.4)	47.3	2.5 ± 2.37	Mild cognitive impairment	186	79.7 (73.1-84.7)	40.9	3.1 ± 2.91
53	Polish	37	67.9 ± 4.5	NA	41.0 ± 26.3	Metabolic syndrome	59	70.9 ± 4.9	NA	45.7 ± 41.4
TNF-alpha (pg/ml) studies										
25 (I)	Spanish	133	87.4 ± 5.3	80.2	8.3 ± 5.8	Sarcopenia (Janssen)	17	87.4 ± 5.3	70.6	7.9 ± 6.2
25 (II)	Spanish	98	87.8 ± 5.0	76.8	9.1 ± 6.2	Sarcopenia (Masanés)	52	87.7 ± 4.9	82.4	6.8 ± 4.7
25 (III)	Spanish	9	86.2 ± 6	70	8.7 ± 4.4	Sarcopenia (low handgrip EWGSOP2)	140	87.8 ± 4.8	79.9	8.2 ± 5.9
46	American	139	61.8 ± 7.6	2.9	5.73 ± 4.84	Barrett’s esophagus	141	62.8 ± 6.7	2.1	5.30 ± 2.72
47	American	925	66 ± 11	19	3.76 ± 2.19	Atrial Fibrillation	46	74 ± 10	6	4.48 ± 2.85
48	American	972	50-79	100	1.4 ± 0.8	Ischemic stroke	972	50-79	100	1.4 ± 0.8
41 (I)	British	698	70.80 ± 5.44	26.9	1.63 ± 0.74	Myocardial infarction	362	70.91 ± 5.48	26.4	1.84 ± 0.84
41 (II)	British	587	71.35 ± 5.24	36.1	1.75 ± 0.77	Stroke	299	71.29 ± 5.32	36.6	1.88 ± 0.79
54	Polish	165	69.21 ± 9.29	46.67	30.83 ± 42.39	Artial fibrillation	80	69.5 ± 8.87	48.7	22.61 ± 29.67
55	Chinese	83	62 ± 10	54.2	65 ± 59	Type 2 diabetes 7 coronary artery disease	237	66 ± 10	32.1	123 ± 115
52	American	1416	71.9 (63.6-78.4)	47.3	4.3 ± 0.89	Mild cognitive impairment	186	79.7 (73.1-84.7)	40.9	3.3 ± 1.64
53	Polish	37	67.9 ± 4.5	NA	72.3 ± 28.5	Metabolic syndrome	59	70.9 ± 4.9	NA	93.2 ± 43.2
IL-1beta (pg/ml) studies										
25 (I)	Spanish	133	87.4 ± 5.3	80.2	1.0 ± 0.7	Sarcopenia (Janssen)	17	87.4 ± 5.3	87.4	1.4 ± 1.5
25 (II)	Spanish	98	87.8 ± 5.0	76.8	1.5 ± 1.7	Sarcopenia (Masanés)	52	87.7 ± 4.9	82.4	1.0 ± 0.8
25 (III)	Spanish	9	86.2 ± 6	70	0.8 ± 0.6	Sarcopenia (low handgrip EWGSOP2)	140	87.8 ± 4.8	79.9	1.4 ± 1.5
46	American	139	61.8 ± 7.6	2.9	0.14 ± 0.13	Barrett’s esophagus	141	62.8 ± 6.7	2.1	0.09 ± 0.15

NA mean no data about division to sex in the analyse.

**Table 3:** The list of publications included in this meta-analysis based on the OR value.

Study	Control				Disesase					OR
	N	Age (years)	% of women	Levels	Type of disease	N	Age (years)	% of women	Levels	
IL-6										
26	622	71.0 (66.0-77.0)	32.5	1.65 ± 1.08	Liver Disease	586	68.0 (64.0-74.0)	80.2	1.58 ± 0.96	4.01 (0.94, 17.14)
44	4532	61.8 ± 10.0)	51.7	1.52 ± 1.21	Intermittent asthma	388	59.7 ± 10.1	60.8	1.60 ± 1.21	1.12 (0.88-1.41)
44	4532	61.8 ± 10.0)	51.7	1.52 ± 1.21	Persistent asthma	109	63.4 ± 9.9	70	1.89 ± 1.61	1.65 (1.08-2.51)
49	68	67.6 ± 11.2	35.3	10.5 ± 17.5	Acute exacerbation	68	70.3 ± 5.8	32.3	35.5 ± 68.3	1.018 (0.999–1.037)
52	1416	71.9 (63.6, 78.4)	47.3	2.5 ± 2.37	Mild cognitive impairment	186	79.7 (73.1-84.7)	40.9	3.1 ± 2.91	1.18 (1.04, 1.34)
52	1416	71.9 (63.6, 78.4)	47.3	2.5 ± 2.37	Mild cognitive impairment	186	79.7 (73.1-84.7)	40.9	3.1 ± 2.91	1.17 (1.01, 1.35)
51	616	85.3 ± 3.3	59	3.25 ± 1.98	Cardiovascular disease	434	86.4 ±4.0	70	3.81 ± 2.80	1.31 (1.11, 1.56)
TNF-alpha										
54	165	69.21 ± 9.29	46.67	22.61 ± 29.67	Artial fibrillation	80	69.5 ± 8.87	48.7	30.83 ± 42.39	0.99 (0.99-1.01)
52	1416	71.9 (63.6-78.4)	47.3	4.3 ± 0.89	Mild cognitive impairment	186	79.7 (73.1-84.7)	40.9	3.3 ± 1.64	1.10 (0.96, 1.25) or 1.09 (0.94, 1.28)
52	1416	71.9 (63.6-78.4)	47.3	4.3 ± 0.89	Mild cognitive impairment	186	79.7 (73.1-84.7)	40.9	3.3 ± 1.64	1.10 (0.96, 1.25) or 1.09 (0.94, 1.28)

### 3.2 Analysis of IL-6 based on SMD

**Figure 2:**
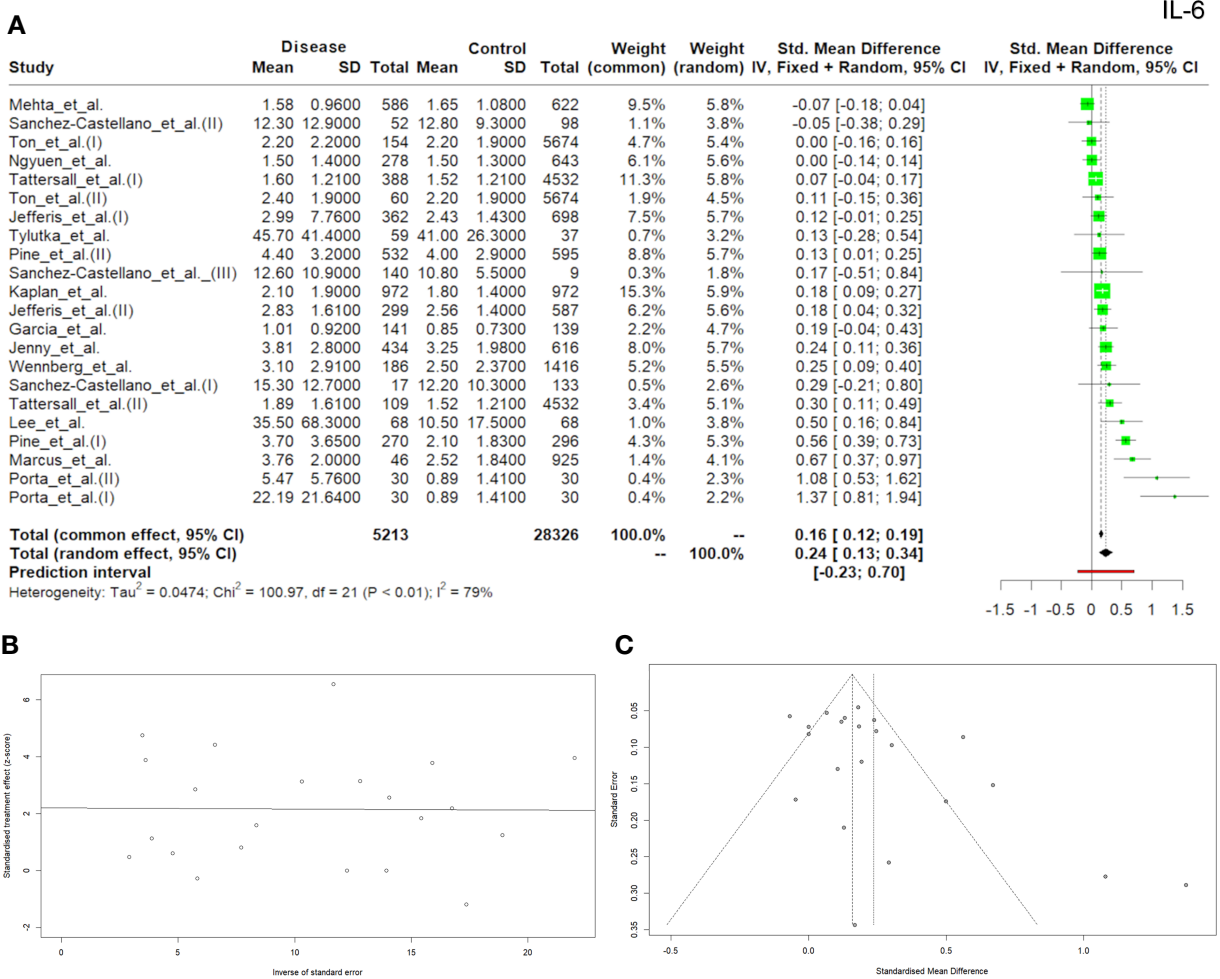
Forrest plot **(A)** Egger test **(B)** and funnel plot **(C)** for IL-6.

Twenty-two studies with the total of 5213 patients with diseases and 28326 controls reported the data on IL-6 concentration. The overall concentration of IL-6 was found to be higher in patients with diseases compared to controls and the difference was significant, with a *p-*value of <0.001 (SMD, 0.16; 95% CI, 0.12–0.19). The heterogeneity was considerable with *Q* = 109.97 (*P <*0.0001) and *I^2 ^= *79.2% (**Figure 2A**). Egger’s test for the publication bias was also significant (*p*=0.02) (**Figure 2B**) and the asymmetry of the funnel plot was observed (**Figure 2C**).

### 3.3 Analysis of TNF based on SMD

**Figure 3:**
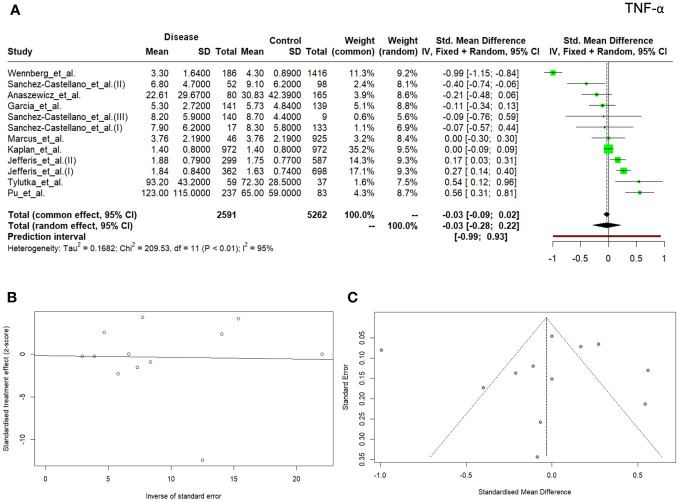
Forrest plot **(A)** and Egger test **(B)** and funnel plot **(C)** for TNF.

The meta-analysis demonstrated that TNF concentration was higher in the controls when compared to patients with diseases (*n* patients = 2591; *n* controls = 5262; SMD, -0.03; 95% CI, -0.09–0.02), however, the value was not significant with *p* – value of 0.533. The observed heterogeneity of the reported studies was high (*Q* =209.53; *P* < 0.0001; *I^2 ^= *94.8%) (**Figure 3A**). Egger’s test for the publication bias was insignificant (*p* = 0.947) (**Figure 3B**), and the asymmetry of the funnel plot was observed (**Figure 3C**). The outliers analysis revealed that two studies had an exceptional impact on the overall effect.

### 3.4 Analysis of IL-1β based on SMD

**Figure 4:**
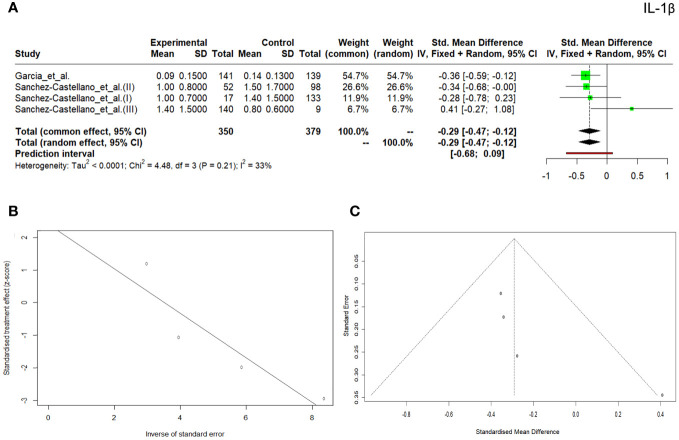
Forrest plot **(A)** and Egger test **(B)** and funnel plot **(C)** for IL-1β.

Three hundred and fifty patients with diseases and 379 controls were included in IL-1β serum concentration meta-analysis which showed a tendency toward increased IL-1β concentration in the control group (SMD, -0.29; 95% CI, -0.47– -0.12; *p* = 0.001). Both overall heterogeneity and Egger’s test for the publication bias were found to be insignificant (*Q* = 4.48; *p=*0.214; *I^2 ^= *33.0% and p =0.213, respectively) (**Figures 4A, B**), but the asymmetry of the funnel plot was observed (**Figure 4C**).

### 3.5 Analysis of IL-6 based on OR

**Figure 5:**
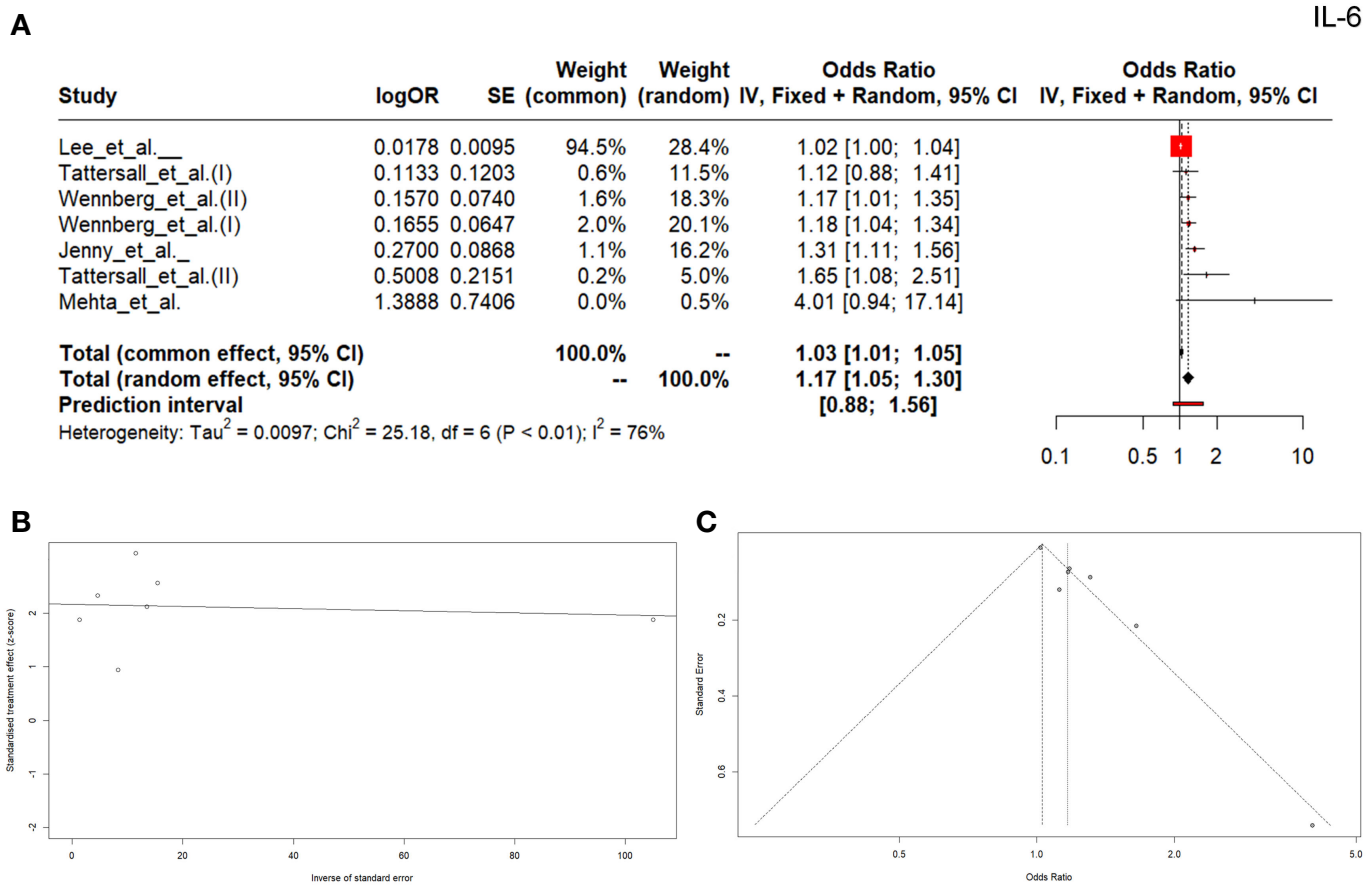
Forrest plot **(A)** Egger test **(B)** and funnel plot **(C)** for IL-6.

The outcomes of seven studies comprising patients and a control group showed OR higher than 1 (OR: 1.03, 95% CI (1.01; 1.05), *p*=0.0029) (**Figure 5A**), which indicates a diagnostic usefulness of the analyzed cytokine. The heterogeneity was Q=25.18, *p*=0.142, I^2 ^= 76.2%. The Egger’s test for the publication bias was found significant (*p*=0.0013, **Figure 5B**), and the asymmetry of the funnel plot was observed (**Figure 5C**).

### 3.6 Analysis of TNF based on OR

**Figure 6:**
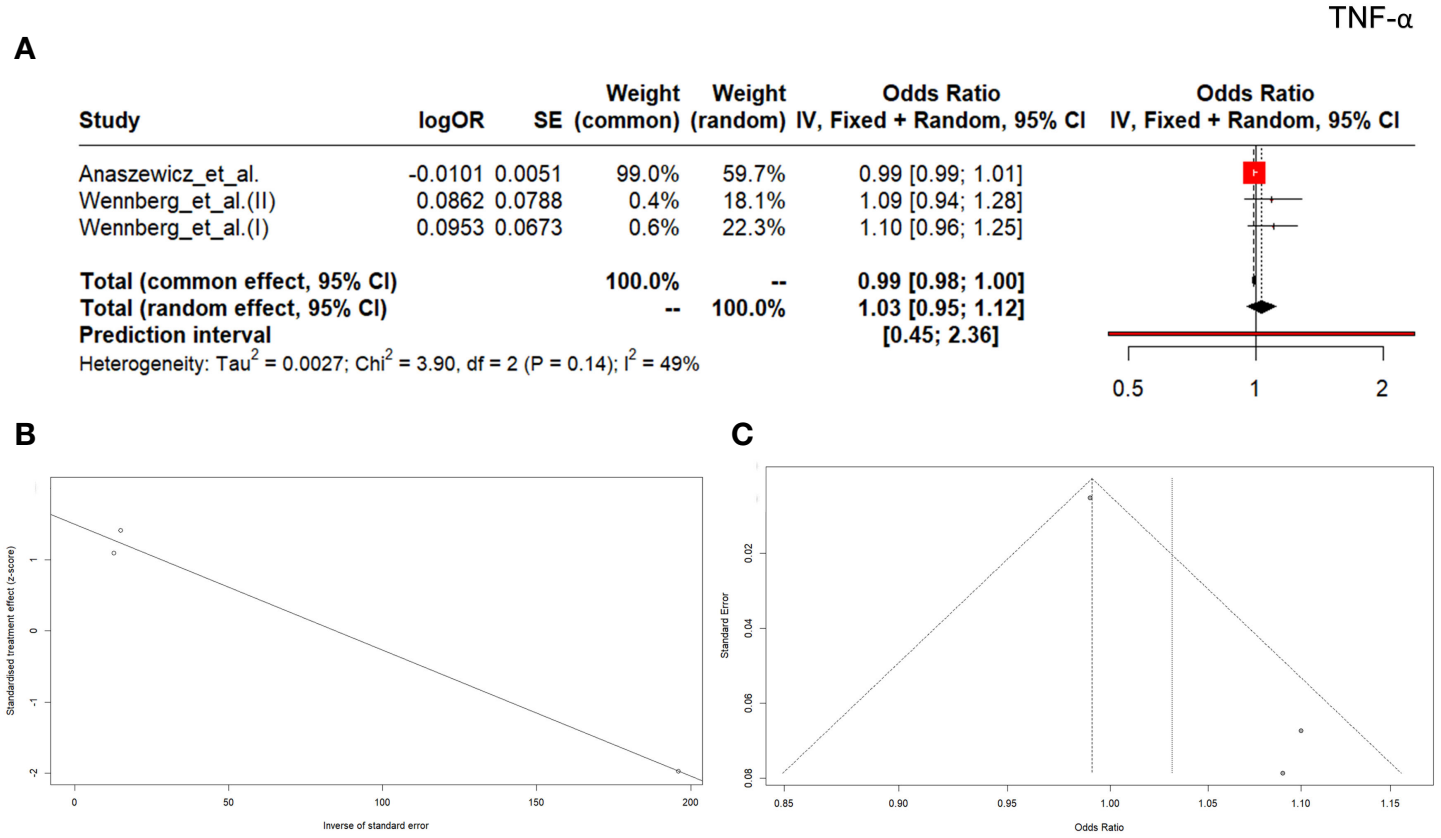
Forrest plot **(A)** and Egger test **(B)** and funnel plot **(C)** for TNF.

The outcomes of three studies including patients showed OR ratio lower than 1 (OR: 0.99, 95% CI (0.98; 1.00), *p* = 0.07) (**Figure 6A**). The heterogeneity was Q=3.90, *p*=0.0003, I^2 ^= 48.7%. The Egger’s test for the publication bias was not significant (*p*=0.08, **Figure 6B**) and the asymmetry of the funnel plot was not observed (**Figure 6C**).

## 4 Discussion

The adaptive immune response weakens with age whereas the innate immune system becomes chronically activated, which is reflected by an increase in the level of pro-inflammatory cytokines. Persistent, low-grade inflammation contributes significantly to the pathophysiology of a variety of age-related conditions and diseases (56). Sex differences in immune response include the production of cytokines that mediate the immune response between cells. Women show greater production of type 2 cytokines, while men show greater production of type 1 cytokines, which include, among others, IL-1β and TNF (57). This analysis and literature review was conducted to analyze the level/concentration of selected cytokines in the elderly. PubMed database papers were selected to assess the level of IL-6, TNF, and IL-1β in a group of elderly people with diverse comorbidities in comparison to a control group of elderly people without coexisting diseases. Significantly increased levels of IL-6 were recorded in patients compared to disease-free group and the outcomes of seven studies showed OR at a slightly higher level than 1. In turn, no statistically significant difference in TNF level was observed between the controls and the patients, whereas a higher concentration of IL-1β was observed in the control group

Most researchers investigating inflammation and aging focus on IL-6 level assessment mainly in the context of the acute phase reaction while there is a growing body of evidence emphasizing the role of this cytokine in the pathogenesis of chronic diseases (58, 59). Research conducted by Xing et al. (60) also suggested that one of the main functions of IL-6 was to self-limit the inflammatory response by inhibiting the production of TNF and IL-1β. IL-6 confines neutrophil recruitment, favoring their replacement by mononuclear cells (61). Consequently, IL-6 may simultaneously regulate both pro-inflammatory and anti-inflammatory activity contributing to both the development/intensification and the suppression of the acute phase reaction. The switch from the inflammatory burst that follows an inflammatory stimulus to the chronic elevation of IL-6 typical of immune-mediated diseases and encountered in many older adults is still much less understood (59). In our meta-analysis, 15 manuscripts met the inclusion criteria. Five publications were evaluated twice, taking into account two groups of patients with diseases vs. one control group, and one manuscript was analyzed three times. A total of 22 records were assessed. Since IL-6 is produced in skeletal muscles and physical activity can cause up to a 10-fold increase in IL-6 levels (62), our meta-analysis excluded the studies which involved any type of intervention (physical activity). Similarly, studies which assessed the effect of diet were also excluded because a high-fat meal increases plasma IL-6 levels (63), which could bias our analyses. In 17 out of 22 papers analyzed, IL-6 levels were higher in the disease group than in the control group with the highest IL-6 levels reported by Tylutka et al. (53), where the patients with the disease (metabolic syndrome) showed an IL-6 value of 45.7 ± 41.4 pg/ml, which was the highest mean value found in all the manuscripts included in the study. This can be explained by the fact that IL-6 produced by adipose tissue accounts for on average 10 to 35% of its basal circulating level in the organism (64). Taking into account the relationship between IL-6 and obesity, it was hypothesized that the main cause of insulin resistance is the increasing level of IL-6 and other pro-inflammatory cytokines (65). Higher mean IL-6 levels in the control group were reported in the studies by Sánchez-Castellano et al. (25), where sarcopenia was analyzed according to the criterion (Masanés), as well as in studies on liver diseases by Mehta et al. (26). In the recorded assessment of Parkinson’s disease and IL-6 levels, neither incident nor prevalent Parkinson’s disease differed significantly compared to the control group (45). No difference in IL-6 levels between diabetic retinopathy (DR) group and control group was shown in the studies by Ngyuen et al. (50). However, the meta-analysis conducted by Yao et al. (66) with 31 case-control studies (groups with and without diabetic retinopathy) demonstrated a relationship between DR and IL-6 level (SMD: 2.12, 95% CI: 1.53-2.70, P<0.00001). This shows that an increased or decreased level of IL-6 depends on the analyzed disease entity, the group of people included in the study and the age or gender of the study subjects. Our meta-analysis showed that IL-6 level in the group of elderly people with various comorbidities was statistically significantly higher compared to the control group with a *p-*value of <0.001 (SMD, 0.16; 95% CI, 0.12–0.19), and obvious heterogeneity existed between the studies *p <*0.0001 and *I^2 ^* = 79.2%. The OR analysis also demonstrated higher values than 1 ​​(OR: 1.03, 95% CI (1.01; 1.05), p=0.0029), which indicates the potential diagnostic usefulness of IL-6 (**Figure 5A**).

TNF is a pro-inflammatory cytokine that affects macrophage functioning and its numerous functions include a key role in the activation of the pro-inflammatory cytokine cascade, which is why it is called the ‘master regulator’ of pro-inflammatory cytokines (67). TNF is an important contributor to tumorigenesis, progression, invasion, and metastasis and it also plays a critical role in autoimmune diseases (68). In our meta-analysis, nine manuscripts were selected for further analyses. One of them was included twice (41) and one underwent our investigation three times (25), which gave us a total of 12 records. Higher values of TNF compared to the control group were noted in the studies conducted on patients with type 2 diabetes and coronary artery disease by Pu et al. (55), as well as in the studies by Tylutka et al. (53), where patients with metabolic syndrome were analyzed. So far, research focused on the relationship between TNF and insulin resistance has provided ambiguous outcomes. Studies on rats conducted by Hotamisligil et al. (69) proved catabolic effect of TNF on adipose tissue and increased peripheral glucose uptake after neutralizing TNF in obese rats. Research on elderly people conducted by Alzamir (33) demonstrated that serum TNF levels in patients with diabetes and obesity were significantly higher than in non-obese counterparts, however, reports by Miyazaki et al. (70) did not confirm such an association. Interestingly, Jefferis et al. (41) showed that TNF was statistically significantly different in the patients with myocardial infarction than in the control group (*p*<0.001), but such a difference was not recorded in the group of patients with stroke vs control group (*p*=0.079), which implied associations between circulating acute phase inflammatory markers and coronary heart disease. Nevertheless, most research papers in our meta-analyses indicated lower TNF values in the group of people with diseases compared to the control, where SMD reached: -0.03; 95% CI, -0.09–0.02, and the value was not significant with *p -*value of 0.533. Additionally, low OR results: 0.99, 95% CI (0.98; 1.00), *p* = 0.07 confirmed our assumption that TNF was a poor predictor in diagnostics.

We also evaluated IL-1β, but only two studies that met the selection criteria were reviewed; therefore, the results should be applied with caution. IL-1β is the best characterized and studied pro-inflammatory cytokine of the 11 members of the IL-1 family. Although most research focuses on its production in monocytes or macrophages it is actually produced by various types of immune cells (71). IL-1β is known to contribute to disease pathogenesis in rheumatoid arthritis, gout, inflammatory bowel disease, and type 2 diabetes (72). More recent evidence highlighted a paradoxical role of IL-1β in diabetic retinopathy, multiple sclerosis, and Alzheimer’s disease (73). Research conducted by Sánchez-Castellano et al. (25) in the group of patients with sarcopenia did not reveal any differences between the groups (regardless of the adopted classification scheme for the study). In turn, the research conducted by Morawin et al. (74) showed statistically significantly higher IL-1β values ​​(*p*=0.02) in the group of patients with sarcopenia *n*=39, 1406.74 ± 1120.23 pg/mL when compared to patients without diagnosed sarcopenia (*n*=134, 755.11 ± 353.90 pg/mL). Our meta-analysis showed higher cytokine values in the control group (SMD, -0.29; 95% CI, -0.47– -0.12; *p* = 0.001), and the overall heterogeneity was not significant (Q = 4.48; p = 0.214; I^2 ^= 33.0%). Since the data on cut-offs and ORs was missing, the diagnostic utility of this cytokine could not be assessed and further research needs to be conducted.

Several shortcomings of this meta-analysis should be acknowledged. Firstly, various disease entities were included in the analyses, which may affect the overall results. Moreover, the individuals included in this meta-analysis have a different geographical nationality. The gender of the examined persons is another point that may affect the analyzed cytokines. Another limitation is related to a disproportion between the size of the control group vs. the group with diseases. Some of the included cytokines lacked enough original studies for the analyses of OR (IL-1β) to be performed. Finally, the analyzed cytokines were determined only in peripheral blood: plasma or serum.

## 5 Conclusion

Our meta-analysis assessed the levels of IL-6, TNF and IL-1β in older adults in relation to disease entities. An increase in IL-6 levels was found in the group with diseases compared to the control group, regardless of the analyzed disease entity. What may suggest a potential role of IL-6 in the progression/development of age-related diseases is the OR result (OR: 1.03, 95% CI (1.01; 1.05), p=0.0029). The evaluation of IL-6 levels may therefore be helpful in the assessment of a disease progression or treatment optimization. Higher values of TNF and IL-1β were found in the control group. Unfortunately, a limited volume of the available scientific literature on IL-1β means that further research is necessary.

## Data availability statement

The raw data supporting the conclusions of this article will be made available by the authors, without undue reservation.

## Author contributions

AT: Conceptualization, Writing – original draft, Writing – review and editing, Methodology. ŁW: Data curation, Formal analysis, Writing – review and editing. AZ-L: Conceptualization, Supervision, Writing – review and editing, Methodology.
