# Supplementary material for: Level of IL-6, TNF, and IL-1β and age-related diseases: a systematic review and meta-analysis
Source: Front Immunol. 2024 Mar 1;15:1330386. doi: 10.3389/fimmu.2024.1330386 (PMC10943692; doi:10.3389/fimmu.2024.1330386)
Supplement: Supplementary file 1 [file DataSheet_1.docx]

Search strategy

 (((("cytokin"[All Fields] OR "cytokine s"[All Fields] OR "cytokines"[Supplementary Concept] OR "cytokines"[All Fields] OR "cytokine"[All Fields] OR "cytokines"[MeSH Terms] OR "cytokinic"[All Fields] OR "cytokins"[All Fields]) AND (("cut"[All Fields] AND "off"[All Fields]) OR ("odds ratio"[MeSH Terms] OR ("odds"[All Fields] AND "ratio"[All Fields]) OR "odds ratio"[All Fields])) AND ("interleukin 6"[Supplementary Concept] OR "interleukin 6"[All Fields] OR "il 6"[All Fields] OR "il6 protein human"[Supplementary Concept] OR "il6 protein human"[All Fields] OR "interleukin 6"[MeSH Terms] OR ("interleukin 6"[Supplementary Concept] OR "interleukin 6"[All Fields] OR "interleukin 6"[All Fields] OR "il6 protein human"[Supplementary Concept] OR "il6 protein human"[All Fields] OR "interleukin 6"[MeSH Terms]) OR ("tumor necrosis factor alpha"[Supplementary Concept] OR "tumor necrosis factor alpha"[All Fields] OR "tnf alpha"[All Fields] OR "tumor necrosis factor alpha"[MeSH Terms] OR ("tumor"[All Fields] AND "necrosis"[All Fields] AND "factor alpha"[All Fields]) OR ("tnf"[All Fields] AND "alpha"[All Fields]) OR ("tumour necrosis factor alpha"[All Fields] OR "tumor necrosis factor alpha"[Supplementary Concept] OR "tumor necrosis factor alpha"[All Fields] OR "tumor necrosis factor alpha"[All Fields] OR "tnf protein human"[Supplementary Concept] OR "tnf protein human"[All Fields] OR "tumor necrosis factor alpha"[MeSH Terms] OR ("tumor"[All Fields] AND "necrosis"[All Fields] AND "factor alpha"[All Fields]) OR ("tumor"[All Fields] AND "necrosis"[All Fields] AND "factor"[All Fields] AND "alpha"[All Fields]))) OR ("interleukin 1beta"[Supplementary Concept] OR "interleukin 1beta"[All Fields] OR "il 1 beta"[All Fields] OR "il1b protein human"[Supplementary Concept] OR "il1b protein human"[All Fields] OR "interleukin 1beta"[MeSH Terms] OR ("interleukin 1beta"[Supplementary Concept] OR "interleukin 1beta"[All Fields] OR "interleukin 1 beta"[All Fields] OR "il1b protein human"[Supplementary Concept] OR "il1b protein human"[All Fields] OR "interleukin 1beta"[MeSH Terms])))) NOT ("education"[MeSH Subheading] OR "education"[All Fields] OR "training"[All Fields] OR "education"[MeSH Terms] OR "train"[All Fields] OR "train s"[All Fields] OR "trained"[All Fields] OR "training s"[All Fields] OR "trainings"[All Fields] OR "trains"[All Fields])) NOT ("therapeutics"[MeSH Terms] OR "therapeutics"[All Fields] OR "therapies"[All Fields] OR "therapy"[MeSH Subheading] OR "therapy"[All Fields] OR "therapy s"[All Fields] OR "therapys"[All Fields])) AND ((ffrft[Filter]) AND (english[Filter]) AND (80andover[Filter] OR aged[Filter]))
